# Infant Feeding Attitudes and Practices of Spanish Low-Risk Expectant Women Using the IIFAS (Iowa Infant Feeding Attitude Scale)

**DOI:** 10.3390/nu10040520

**Published:** 2018-04-22

**Authors:** María del Carmen Suárez Cotelo, María Jesús Movilla-Fernández, Paula Pita-García, Silvia Novío

**Affiliations:** 1 Research Group GRINCAR, Obstetrics Department, Hospital Álvaro Cunqueiro, C/Clara Campoamor, 341, 36312 Vigo, Pontevedra, Spain; 2 Research Group GRINCAR, Department of Health Sciences, Faculty of Nursing and Podiatry, University of A Coruña, Campus de Esteiro, C/ Naturalista López Seoane, s/n (esquina San Ramón), 15403 Ferrol, A Coruña, Spain; maria.jesus.movilla@udc.es; 3 Obstetrics Department, Hospital Arquitecto Marcide-Novoa Santos, Avda. de la Residencia s/n, 15405 Ferrol, A Coruña, Spain; paulapitagarcia@gmail.com; 4 Department of Psychiatry, Radiology, Public Health, Nursing and Medicine; Faculty of Nursing, University of Santiago de Compostela, C/San Francisco, s/n, 15782 Santiago de Compostela, A Coruña, Spain

**Keywords:** attitude, breastfeeding, Iowa Infant Feeding Attitude Scale, Spain, validity

## Abstract

The Iowa Infant Feeding Attitude Scale (IIFAS) has been shown to have good psychometric properties for English-speaking populations, but it has not been validated among low-risk pregnant women in Spain. The aim of this study was to assess the reliability and validity of the translated version of the IIFAS in order to examine infant feeding attitudes in Spanish women with an uncomplicated pregnancy. Low-risk expectant women (*n* = 297) were recruited from eight primary public health care centres in Galicia (Spain). Questionnaires including both socio-demographic and breastfeeding characteristics and items about infant feeding were administered during the third trimester. Participants were contacted by telephone during the postpartum period to obtain information regarding their infant feeding status. Prediction validity and internal consistency were assessed. The translated IIFAS (69.76 ± 7.75), which had good psychometric properties (Cronbach’s alpha = 0.785; area under the curve (AUC) of the receiver operating characteristic (ROC) curve = 0.841, CI_95%_ = 0.735–0.948), showed more positive attitudes towards breastfeeding than towards formula feeding, especially among mothers who intended to exclusively breastfeed. This scale was also useful for inferring the intent to breastfeed and duration of breastfeeding. This study provides evidence that the IIFAS is a reliable and valid tool for assessing infant feeding attitudes in Spanish women with an uncomplicated pregnancy.

## 1. Introduction

Breast milk is recognized worldwide as the optimal food for newborns as it confers substantial health advantages to both the child and the mother [1]. Exclusive breastfeeding for the first 6 months of life is recommended, with the gradual introduction of complementary foods and continuation of breastfeeding up to 2 years of age or beyond [2]. However, women in many countries do not follow these recommendations. In this respect, the most recent statistics in Spain show that the rates of breastfeeding (exclusive and partial) are 72.4% at 6 weeks postpartum, 66.5% at 3 months postpartum and 47% at 6 months postpartum [3]. 

Psychosocial factors, such as maternal attitudes about infant feeding, have been found to be better predictors of feeding methods compared to sociodemographic factors [4]. Chen and Chi [5] have shown that the maternal positive attitudes towards breastfeeding are associated with continued breastfeeding at the first postpartum month. Therefore, it is important to assess women’s intentions in order to choose strategic approaches for increasing initiation and duration rates.

The Iowa Infant Feeding Attitude Scale (IIFAS), developed by De la Mora and Russell [6], has been found to be a reliable and valid instrument to assess women’s attitudes regarding infant feeding and to predict the choice of feeding methods and duration of breastfeeding in diverse populations and in a number of countries [4,6,7,8,9,10,11,12,13,14,15,16,17,18,19,20,21,22,23,24,25,26,27,28]. Furthermore, its use was supported by a systematic review [29]. 

Tomas-Almarcha et al. [24] have recently translated and validated a Spanish version of IIFAS (Simplified Spanish) in expectant women recruited from hospitals in Eastern Spain. According to clinical practice guidelines in the Spanish National Health System [30], the sample was constituted of high-risk pregnant women. Taking into account that breastfeeding behaviours depend on pregnancy complexity [31], this Spanish version of IIFAS should also be validated in women with uncomplicated pregnancies. 

This study aimed: (a) to describe the development and validation of a semantically and culturally appropriate Spanish version of the IIFAS among low-risk pregnant women in Spain; and (b) to examine infant feeding attitudes in this sample. 

## 2. Materials and Methods

### 2.1. Subjects and Setting

The study was conducted between June 2014 and February 2016 in the Health Area of Ferrol, which is one of the 13 Galician Health Areas (2009–2013 average annual labours = 1231). This area has 22 primary public health care centres, 16 of which have a midwife service. These health care centres serve populations with different levels of socio-economic status and antenatal care provided by them is representative of antenatal care given to low-risk pregnant women in Galicia, which is an Autonomous Region of Spain with two official languages (Spanish and Galician).

A sample of 297 women with an uncomplicated pregnancy between 26 and 38 weeks of gestation were recruited from all primary public health care centres of this health area by midwives, who were willing to participate in the study. The sample size was determined considering a population of 1231 pregnant women (2009–2013 average annual labours), with an expected prevalence of exclusive breastfeeding at 6 months of 5%, with a 95% of confidence level and 3% of precision. The loss-to-follow-up rate was anticipated to be 15%.

**Scheme 1:**
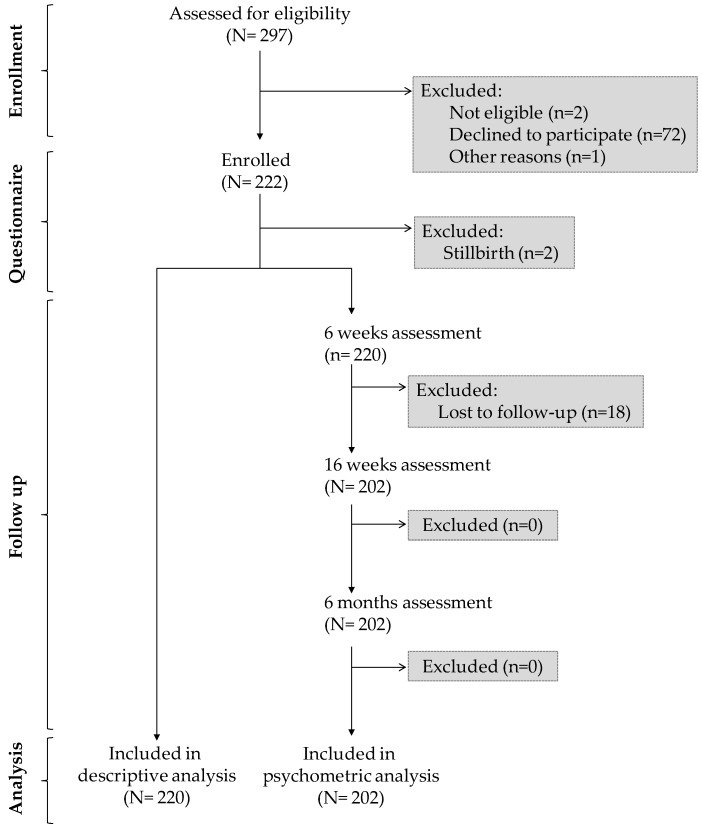
Flow chart of study population selection. The grey boxes show the women excluded from the study.

Participants, who met the following inclusion criteria, were invited to participate in the study: (1) low-risk pregnant women attending childbirth education classes with the midwife in any of the centres previously mentioned; (2) they were able to read and write in Spanish or Galician and understood the survey directions and questions; (3) they were 18 years of age or older; and (4) they had a single pregnancy as determined by obstetricians. The participants recruited initially were excluded from the study if the pregnancy ended in either miscarriage or stillbirth or if breastfeeding data were unavailable at follow-up interviews at 6 weeks, 16 weeks and 6 months. Two midwives were trained in recruitment, procedures and interviewing. All eligible women were approached and recruited on the day they attended childbirth education classes (Scheme 1). No differences, in terms of sociodemographic characteristics, were found among them. 

The study has been carried out in accordance with the Declaration of Helsinki and approved by the Comité Autonómico de Ética de la Investigación de Galicia (CAEIG) under the protocol number 2014/064. Likewise, all women gave written consent for participation in the study after being provided with adequate information about the purposes of the study.

### 2.2. Data Collection

Spanish or Galician questionnaires were delivered by hand to all eligible women at the end of a childbirth education class. If mothers were willing to participate in the study, they returned the enclosed questionnaire by post to the principal investigator. 

The questionnaire consisted of two sections. The first section included data on participants’ socio-demographic (age, nationality, level of education, occupation, marital status, parity and primary public health care centre) and breastfeeding characteristics (previous breastfeeding experience and infant feeding intention). The second one assessed women’s infant feeding attitudes using the IIFAS (Table A1). IIFAS-t (IIFAS translated) was the terminology used to refer to both versions of IIFAS [Spanish version (IIFAS-S) and Galician one (IIFAS-G)]. IIFAS consists of 17 items that are scored on a 5-point Likert scale, which ranges from 1 (strongly disagree) to 5 (strongly agree). Eight of the items are worded in a favourable manner towards breastfeeding, while the remaining 9 are favourable towards formula-feeding. Items favouring formula-feeding are reverse-scored (i.e., 1 = 5, 2 = 4, 4 = 2 and 5 = 1), and a total of attitude score is computed via an equally weighted sum of responses to the individual items. The total attitude scores range from 17 (indicating positive bottle-feeding attitudes) to 85 (reflecting positive breastfeeding attitudes) [6]. Total scores are grouped into the following three categories: (1) positive towards breastfeeding (70–85); (2) neutral (49–69); and (3) positive towards formula feeding (17–48) [27]. 

To follow up on infant feeding status, participants were phoned at 6 weeks, 16 weeks and 6 months postpartum. The women were asked how they were feeding their baby at home and their method of feeding was classified as: exclusive breastfeeding or non-exclusive breastfeeding (partial breastfeeding or formula feeding).

### 2.3. Development and Clinical Validation of the Spanish Version of the IIFAS (IIFAS-S)

The translation and validation of the IIFAS for use in Spain were authorized by the author of the original instrument, Dr. De La Mora. 

#### 2.3.1. Development of the Spanish Version of the IIFAS (IIFAS-S)

The cross-cultural adaptation process followed the steps outlined by Beaton et al. [32]. The method developed by Sperber et al. [33] was used for establishing semantic equivalence and validating the translated instrument. Each item in the original and back-translated versions was ranked in terms of comparability of language and similarity of interpretability. Likert scales ranging from 1 (extremely comparable/extremely similar) to 7 (not at all comparable/not at all similar) were used for ranking by participants, who were fluent in English. Any mean score >3 required a formal review of the translation. After several minor changes, the Spanish translation was deemed to be semantically equivalent to the original version. A pilot study was conducted with 22 pregnant women to test the comprehensibility and legibility of the IIFAS-S. No problem of comprehension was detected by participants, so no item was changed. 

#### 2.3.2. Clinical Validation of the Spanish Version of the IIFAS (IIFAS-S)

After translation and adaptation, statistical analyses were performed to evaluate the psychometric properties of the translated instrument, with a focus on its clinical validity, which is namely its ability to assess what it was designed to measure [32]. We analysed cross-sectional data collected during the prenatal and postpartum period to determine the reliability and validity of the IIFAS-S in a population of low-risk pregnant Spanish women in terms of breastfeeding intention and duration. Participants were asked in the questionnaire (prenatal period) and by phone (postpartum period): “How will you feed your new baby?” and “How have you fed your new baby?”. The response variables were “I have decided to exclusively breastfeed” and “I have decided to (fully or partially) formula feed”. For the purpose of analysis, when the answer during prenatal period was “I have not decided it yet”, as the input was no intention to breastfeed. These variables served as the testing variables (dependent variables) in our current study for the validation of the IIFAS-S [26]. 

### 2.4. Statistical Methods

The IBM Statistical package for Social Sciences, Version 20.0 (SPSS for Windows, SPSS Inc., Madrid, Spain) was used to analyse the data. *p* values < 0.05 were considered to be significant.

#### 2.4.1. Descriptive Analysis

Records with >50% missing variables were removed (*n* = 2). Missing values were assumed to be missing at random and were used as inputs using a regression estimate of the missing value based on all observations. 

Descriptive statistics and cross-tabulations were employed to summarize the characteristics of the sample and each item in the IIFAS-t. 

The Chi-square test was used to compare categorical variables (socio-demographic and breastfeeding characteristics).

#### 2.4.2. Attitudes towards Breastfeeding

The Chi-square test was used to: (i) assess how the IIFAS-t total score relates to socio-demographic factors; and (ii) assess the differences in mothers’ infant-feeding attitudes in relation to feeding intention (exclusively breastfeed/not exclusively breastfeed [fully or partially formula feed]) and breastfeeding duration (exclusively breastfeed/not exclusively breastfeed [fully or partially formula feed] at 6 weeks, 16 weeks and 6 months). Furthermore, bivariate logistic regression was used to determine the relationship between the socio-demographic factors and the positive attitude towards breastfeeding.

#### 2.4.3. Psychometric Analysis

The corrected item total correlation, the Cronbach’s alpha coefficient (α*) if the item is deleted and the overall Cronbach’s alpha coefficient (α) were calculated to evaluate the reliability of the total IIFAS-S in the study sample of low-risk expectant women. The validity of the total IIFAS-S score was examined using the receiver operating characteristic (ROC) curve. The area under the graph (0.841 mean with a 95% confidence interval (CI) of 0.735–0.948) was assessed for its sensitivity and specificity of the total IIFAS-S score in predicting intent to breastfeed. 

Finally, the predictive validity was determined by examining the association between: (i) the IIFAS-S score during pregnancy and intention of exclusive breastfeeding; and (ii) the IIFAS-S score during pregnancy and duration of exclusive breastfeeding at 6 weeks, 16 weeks and 6 months postpartum. It was analysed by Chi-square tests and bivariate logistic regression.

## 3. Results

### 3.1. Sample Description

In total, 297 women were eligible to participate and 220 completed the questionnaire (response rate of 75%). Of those that filled the questionnaire, 122 filled in the Spanish questionnaire and 98 filled in the Galician one. Socio-demographic and breastfeeding characteristics of the population enrolled were compared according to the language (Table 1). There were no significant differences for the characteristics measured between 2 groups (*p* > 0.05). The majority of women were 18–34 years of age, Spanish and married. One-third of the women had completed secondary education with 43.6% having studied at university. Most of the women had a paid job (71.8%). From the 220 women, 22.3% of women were multiparous. Most of the women (89.8%) had breastfed previously, consisting of 20.0% of the total subject group. 

### 3.2. Attitudes towards Breastfeeding

The IIFAS summary scores had normal distributions, which was assessed by Kolmogorov–Smirnov test (*p* > 0.05, data not shown) and normality statistics (Table A2). 

Table 2 shows the women’s attitudes towards breastfeeding using IIFAS-t. The average score of the IIFAS-t (mean ± standard deviation) was 69.76 ± 7.75. The items with the lowest and highest scores were 17 and 16, respectively.

### 3.3. Infant-Feeding Attitudes and Demographic Factors

Women who were multiparous had higher IIFAS-t scores, indicating more favourable attitudes towards breastfeeding. On the contrary, there were no differences in regard to marital status, age, education level and occupation (Table 3). Likewise, when bivariate logistic regression was undertaken with the positive attitude towards breastfeeding set as the dependent variable, the results showed that IIFAS-t total scores ≥70 were more likely among women who were multiparous (adjusted OR = 2.653, 95% CI = 1.172–5.988).

### 3.4. Validity

#### 3.4.1. Predictive Validity

##### Prediction of breastfeeding intention

The majority of participants intended to exclusively breastfeed (90%), while 9.6% intended to formula-feed or to partially breastfeed (Table 1). There were significant differences (*p* = 0.000) in total IIFAS scores between women who intended to exclusively breastfeed (70.75 ± 6.97) and those who did not (59.90 ± 7.70). In general, the higher scores on all 17 items individually showed a positive association with intention to breastfeed, although they were not statistically significant in all cases (Table 4). 

**Figure A1:**
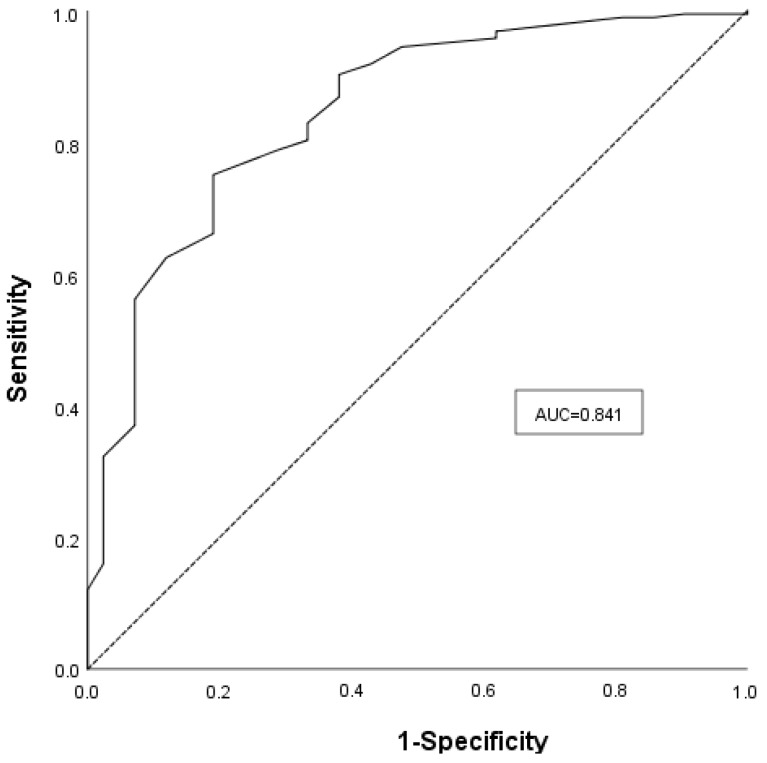
Receiver Operating Characteristic (ROC) curve. Abbreviation: AUC = area under the curve.

In order to assess the validity of the IIFAS-S in predicting the intention of breastfeeding, the area under the curve (AUC) for the ROC curve was analysed (Figure A1) and it was found to be 0.841 (CI_95%_ = 0.735–0.948). Therefore, we can state that the IIFAS-S is valid in predicting breastfeeding intent in low-risk Spanish pregnant women.

##### Prediction of breastfeeding duration

Out of 113 women, 63 (55.8%) continued with exclusive breastfeeding at 6 weeks, 50 (44.2%) at 16 weeks and 25 (22.1%) at 6 months. There were significant differences in total IIFAS scores between women with exclusive breastfeeding at 6 weeks (71.10 ± 6.87), 16 weeks (71.50 ± 7.12) or 6 months (72.17 ± 6.47) compared to those with non-exclusive breastfeeding (partial or no breastfeeding) (66.92 ± 8.74 at 6 weeks, 67.52 ± 8.08 at 16 weeks and 67.79 ± 8.22 at 6 months; Table 5).

The duration of exclusive breastfeeding was higher among women who intended to exclusively breastfeed (Table 6). When bivariate logistic regression was undertaken with the duration of breastfeeding set as the dependent variable, the results showed that exclusive breastfeeding was more likely among mothers, who had previous breastfeeding experience at 6 weeks [adjusted OR =3.175, 95% CI = 1.677–8.411], 16 weeks [adjusted OR = 2.793, 95% CI = 1.844–7.633] and 6 months [adjusted OR = 2.245, 95% CI = 1.511–5.703) or were multiparous (16 weeks [adjusted OR = 8.022, 95% CI = 5.004–9.177]).

#### 3.4.2. Internal Consistency Reliability

The Cronbach’s alpha of the IIFAS-S questionnaire was α = 0.785. The psychometric data for the assessment of the reliability and internal consistency of the IIFAS items are shown in Table A3. The corrected item total correlations were all positive in the range of 0.127–0.588 for the Spanish sample. Likewise, the Cronbach’s α* for each item deleted remained above 0.76, demonstrating the reliable use of the IIFAS in the low-risk prenatal population of Spain.

## 4. Discussion and Conclusions

To our knowledge, this is the first longitudinal study to describe the development and psychometric properties of the extended version of the IIFAS-S and its use in predicting the breastfeeding intention and duration in a sample of Spanish woman with an uncomplicated pregnancy. These data provide evidence that this version of the IIFAS is a reliable and valid tool to understand infant feeding practises in low-risk expectant Spanish mothers. In this way, the IIFAS showed more positive attitudes towards breastfeeding than towards formula feeding.

The IIFAS-S had reasonable reliability and validity. The Cronbach’s alpha in the present study falls into Devellis’s [34] “respectable” range, indicating acceptable reliability. This is comparable to values observed in other studies with samples of expectant mothers (α = 0.66–0.87) [4,18,22,24,26], except for the study carried out by Wallis et al. [27] where α = 0.50. Furthermore, the reliability of the instrument is strengthened by the normality of the distributions [35] (Table A2). On the other hand, in relation to the validity, the finding that women who expressed the intention to breastfeed exclusively during prenatal period had a more positive attitude towards breastfeeding than the women who did not express this intention supports the construct validity of the scale.

Recently, the need to explore item reduction for the Spanish version of the IIFAS has been suggested [24]. It would have been interesting to have evaluated the psychometric equivalence between the complete and short versions, because sometimes incomplete equivalences are discovered too late [36]. Likewise, the reliability and validity of short versions of scales have been questioned [37,38]. In our study, statistics on the psychometric properties do not maintain this need (α = 0.785, item-total correlations higher than 0.30, ...) and thus, the IIFAS-S is ready to be used in studies assessing maternal infant feeding attitudes in low-risk expectant Spanish mothers.

The association of the IIFAS total score with socio-demographic factors has been previously studied [12,19,22]. However, up to now, no research has observed different IIFAS scores according to parity. Although more information is needed, we suggest that the higher scores observed among multiparous women may be due to these women having received more information and having had more time to think about feeding methods due to their previous experience with other pregnancies.

In the present study, we have observed a more positive maternal attitude towards breastfeeding than that observed in two previous studies carried out by Scott et al. [39] and Tomas-Almarcha et al. [24] in a sample of Spanish women. In principle, this is particularly striking considering that the latter recruited a convenience sample from hospitals, which were implementing strategies to improve breastfeeding practices or were Baby-Friendly hospitals. Although it is not possible to know this for certain, this divergence of results could be due to differences in the translation and/or sample characteristics.

In relation to mean item responses (Table 2), the following aspects must be highlighted: (1) there is an inconsistency between the items 5 and 10, which might be explained by the common Spanish culture belief that “gaining weight and being fat means prosperity”; (2) the high mean score of the item 8 might mean the approval of women’s rights to breastfeed in public, although this perception does not reflect the opinion of all the population (e.g., ≥45 years) [40]; (3) the low mean score of the item 17 indicates that more information is required about the compatibility of breastfeeding and occasional consumption of alcohol [41]; and (4) the high mean score of the item 6 might reflect the support that Spanish mothers receive for breastfeeding in the workplace [42], despite most of the women considering it to be insufficient for continuing exclusive breastfeeding on demand for 6 months [43]. In this respect, the prevalence of breastfeeding (exclusive or partial) observed in the present study (82.84% at 6 weeks, 70.1% at 16 weeks, 57.35% at 6 months) in comparison to the prevalence that was reported in our country 4 years ago [3] seems to support this idea.

According to the predictive validity of the IIFAS-S, it could be used by primary health-care professionals in order to understand general attitudes, to pinpoint specific attitudes or gaps in knowledge and to identify women slightly receptive to breastfeeding. This information could contribute to both the design of breastfeeding promotional interventions (e.g., programs, campaigns) and policies that specifically target relevant issues where the infant-feeding attitudes of mothers are poor and the evaluation of these interventions/policies once they are implemented (pre-/post-test tool).

Our study included several limitations. First, the majority of participants were not unemployed, so probably this sample would not have budget concerns. Second, we have studied a clinic sample, which might represent a group that is more compliant and better informed about infant feeding methods than a random sample. Likewise, the context of the research might have primed participants to give what they perceived to be socially desirable answers, especially in the questions of items 6 and 8. In order to reduce this bias, all respondents were assured that their participation would not affect their health care and the information provided would be held in confidence. Third, as participants filled in questionnaires themselves, there may be some self-report bias. Because of these limitations, additional studies are needed to determine if the results from our study can be generalized to low-risk expectant Spanish women with other characteristics (e.g., women who live in big cities and those who are socioeconomically disadvantaged).

Our research provides evidence that the IIFAS is a reliable and valid tool for assessing infant feeding attitudes in Spanish women with an uncomplicated pregnancy. This suggests that the Spanish version could contribute to both the design and evaluation of breastfeeding promotional interventions and policies by primary health-care professionals.

## Figures and Tables

**Table 1:** Characteristics of the study population (*n* = 220).

		Spanish (*n* = 122)		Galician (*n* = 98)		Total (*n* = 220)	
		*n*	%	*n*	%	*n*	%
Mother’s age	18–34	73	59.8	63	64.3	136	61.8
	≥35	48	39.3	35	35.7	83	37.7
	No response	1	0.8	0	0	1	0.5
Marital status	Married	69	56.6	56	57.1	125	56.8
	Cohabiting	45	36.9	39	39.8	84	38.2
	Single	7	5.7	2	2.0	9	4.1
	No response	1	0.8	1	1	2	0.9
Nationality	Spanish	116	95.1	97	99	213	96.8
	Others	6 ^a^	4.9	1 ^b^	1	7	3.2
	No response	0	0	0	0	0	0
Level of education	Secondary education or lower	45	36.9	29	29.6	74	33.6
	Apprentice	22	18	26	26.5	48	21.8
	Graduate or above	54	44.3	42	42.9	96	43.6
	No response	1	0.8	1	1.0	2	0.9
Parity	Primiparous	100	82.0	71	72.4	171	77.7
	Multiparous	22	18.0	27	27.6	49	22.3
	No response	0	0	0	0	0	0
Primary public health care centre	Ferrol	49	40.2	32	32.7	81	36.8
	Narón	48	39.3	38	38.8	86	39.1
	Fene	11	9.0	14	14.3	25	11.4
	Ortigueira	2	1.6	1	1	3	1.4
	As Pontes de García Rodríguez	8	6.6	10	10.2	18	8.2
	Valdoviño	3	2.5	2	2	5	2.3
	San Sadurniño	1	0.8	1	1	2	0.9
	No response	0	0	0	0	0	0
Occupation	Employee	80	65.6	60	61.2	140	63.6
	Self-employed	10	8.2	8	8.2	18	8.2
	Student	4	3.3	3	3.1	7	3.2
	Housewife	27	22.1	27	27.6	54	24.5
	No response	1	0.8	0	0	1	0.5
Previous breastfeeding experience ^c^	Exclusive breastfeeding	14	63.6	16	59.3	30	61.2
	Fully or partially formula feed	8	36.4	9	33.3	17	34.7
	No response	0	0	2	7.4	2	4.1
Infant feeding intention	Exclusive breastfeeding	106	86.9	92	93.9	198	90
	Fully or partially formula feed	15	12.3	6	6.1	21	9.6
	It had not been decided	1	0.8	0	0	1	0.5

Statistical differences were not noted between women who filled in the Spanish questionnaire and the Galician one (Chi square; * *p* < 0.05). ^a^ Portuguese (*n* = 1), Romanian, Colombian (*n* = 3), Polish (*n* = 1), Peruvian (*n* = 1); ^b^ Portuguese (*n* = 1); ^c^ Calculated among those who had previous children.

**Table 2:** Mothers’ attitudes towards breastfeeding using the IIFAS-t.

Item Variable ^a^	M	SD	Agree (%)	Neutral (%)	Disagree (%)
1. The nutritional benefits of breast milk last only until the baby is weaned from breast milk ^b^	3.95	1.27	73.6	8.6	17.7
2. Formula feeding is more convenient than breast-feeding ^b^	4.64	0.75	93.2	4.1	2.7
3. Breast-feeding increases mother–infant bonding	4.64	0.83	93.6	2.3	4.1
4. Breast milk is lacking in iron ^b^	4.26	0.98	82.7	10.9	6.4
5. Formula fed babies are more likely to be overfed than breast-fed babies	3.32	1.16	46.8	28.2	25
6. Formula feeding is the better choice if a mother plans to work outside the home ^b^	3.81	0.96	65.5	25.9	8.6
7. Mothers who formula feed miss one of the great joys of motherhood	3.80	1.11	61.4	28.2	10.5
8. Women should not breast-feed in public places such as restaurants ^b^	4.40	0.98	84.1	10.9	5.0
9. Babies fed breast milk are healthier than babies who are fed formula	3.95	1.08	70.0	18.2	11.8
10. Breast-fed babies are more likely to be overfed than formula fed babies ^b^	3.98	1.05	72.7	19.1	8.2
11. Fathers feel left out if a mother breast-feeds ^b^	4.18	0.88	82.7	12.3	5
12. Breast milk is the ideal food for babies	4.63	0.78	93.6	3.6	2.7
13. Breast milk is more easily digested than formula	4.24	0.91	77.3	19.5	3.2
14. Formula is as healthy for an infant as breast milk ^b^	4.00	0.91	77.7	15.0	7.3
15. Breast-feeding is more convenient than formula feeding	4.47	0.78	89.1	8.6	2.3
16. Breast milk is less expensive than formula	4.75	0.63	95.9	2.3	1.8
17. A mother who occasionally drinks alcohol should not breast-feed her baby ^b^	2.74	1.26	33.2	19.1	47.7
Total	69.76	7.75	57.7	40.9	1.4

^a^ Participants (*n* = 216) were asked how they agreed with each statement of a 5-point Likert-type scale ranging from 1 (strongly disagree) to 5 (strongly agree). In turn, these scores were grouped into the following three categories: disagree/positive towards formula feeding (scores 1 and 2), neutral (score 3) and agree/positive towards breastfeeding (scores 4 and 5). ^b^ This was reversed when calculating the score. Abbreviations: M = means; and SD = standard deviations.

**Table 3:** Differences in attitudes towards breastfeeding by demographic factor, as determined by Iowa Infant Feeding Attitude Scale scores (IIFAS-t). IIFAS scores range from 17 to 85 with higher scores reflecting more positive attitudes on breastfeeding.

Demographic Factor ^a^		Mean Score (SD)	*p*
Mother’s age	18–34	68.77 (7.93)	0.112
	≥35	71.35 (7.25)	
Marital status	Married	70.06 (8.11)	0.101
	Cohabiting	69.89 (6.35)	
	Single	65.67 (12.55)	
Level of education	Secondary education or lower	69.46 (7.82)	0.699
	Apprentice	68.79 (8.32)	
	Graduate or above	70.60 (7.32)	
Parity	Primiparous	68.80 (7.70)	0.034 *
	Multiparous	73.10 (7.05)	
Occupation	Employee	69.76 (8.17)	0.798
	Self-employed	69.67 (7.73)	
	Student	68.43 (4.35)	
	Housewife	69.91 (7.15)	

Statistical differences were noted with * *p* <0.05 (Chi-square test). ^a^ Sample size, *n* = 216.

**Table 4:** Assessment of validity of IIFAS-S items and total scores by intent to breastfeed.

	Exclusively Breastfeed						
	Yes			No			
Item Variable ^a,b^	Agree (%)	Neutral (%)	Disagree (%)	Agree (%)	Neutral (%)	Disagree (%)	*p* Value
1 ^c^	74.5	8.5	17	66.7	6.7	26.7	0.657
2 ^c^	95.3	1.9	2.8	73.3	26.7	0	0.000
3	95.3	1.9	2.8	73.3	13.3	13.3	0.009
4 ^c^	85.8	9.4	4.7	80.0	20.0	0.0	0.345
5	49.1	29.2	21.7	26.7	20.0	53.3	0.031
6 ^c^	65.1	27.4	7.5	33.3	46.7	20	0.048
7	69.8	25.5	4.7	33.3	20.0	46.7	0.000
8 ^c^	85.8	11.3	2.8	46.7	33.3	20.0	0.001
9	75.5	17	7.5	13.3	33.3	53.3	0.000
10 ^c^	68.9	20.8	10.4	86.7	13.3	0.0	0.285
11 ^c^	87.7	9.4	2.8	73.3	13.3	13.3	0.134
12	95.3	2.8	1.9	66.7	33.3	0.0	0.000
13	83.0	16.0	0.9	53.3	40.0	6.7	0.018
14 ^c^	82.1	14.2	3.8	46.7	20.0	33.3	0.000
15	89.6	8.5	1.9	66.7	20.0	13.3	0.021
16	93.4	4.7	1.9	100	0	0	0.591
17 ^c^	27.4	21.7	50.9	20.0	6.7	73.3	0.224
Total score	59.4	40.6	0.0	13.3	73.3	13.3	0.000

Mean difference for individual IIFAS-S items and total scores by intent to breastfeed were estimated. ^a^ The name of the items can be found in Table 2 and Table A1. ^b^ Sample size, *n* = 216. ^c^ This was reversed when calculating the score.

**Table 5:** Assessment of validity of IIFAS-S total scores by duration of breastfeeding. The relationship between the duration of breastfeeding and the IIFAS total scores was examined. IIFAS total score was significantly associated with exclusive breastfeeding at 6 and 12 weeks and 6 months postpartum.

		Exclusively Breastfeed ^a^						
		Yes			No			
	Duration	Agree (%)	Neutral (%)	Disagree (%)	Agree (%)	Neutral (%)	Disagree (%)	*p* Value
IIFAS	6 weeks	38 (60.3)	25 (39.7)	0 (0.0)	23 (46.0)	25 (50.0)	2 (4.0)	0.019
	16 weeks	32 (64.0)	18 (36.0)	0 (0.0)	29 (46.0)	32 (50.8)	2 (3.2)	0.049
	6 months	17 (68.0)	8 (32.0)	0 (0.0)	44 (50.0)	42 (47.7)	2 (2.3)	0.024

Participants were asked how they agreed with each statement of a 5-point Likert-type scale ranging from 1 (strongly disagree) to 5 (strongly agree). In turn, these scores were grouped into the following three categories: disagree/positive towards formula feeding (scores 1 and 2), neutral (score 3) and agree/positive towards breastfeeding (scores 4 and 5). Mean difference for total scores by duration of breastfeeding were estimated. ^a^ Sample size, n = 113.

**Table 6:** Breastfeeding intentions among participants who initiated exclusive breastfeeding compared to those who did not at 6 weeks, 16 weeks and 6 months.

		Exclusively Breastfeeding *n* (%)	Non-Exclusively Breastfeeding *n* (%)	*p*
6 WEEKS				
Intention	Exclusively breastfeeding	60 (60.6)	39 (39.4)	0.006
	Nonexclusively breastfeeding	3 (21.4)	11 (78.6)	
16 WEEKS				
Intention	Exclusively breastfeeding	48 (48.5)	51 (51.5)	0.016
	Nonexclusively breastfeeding	2 (14.3)	12 (85.7)	
6 MONTHS				
Intention	Exclusively breastfeeding	24 (24.2)	75 (75.8)	0.049
	Nonexclusively breastfeeding	1 (7.1)	13 (92.9)	

## Appendix A

**Table A1:** IIFAS (Iowa Infant Feeding Attitude Scale) items. Spanish and English versions.

Spanish Version	English Version
1. Los beneficios nutricionales de la leche materna únicamente se mantienen hasta que el bebé es destetado.	1. The nutritional benefits of breast milk last only until the baby is weaned from breast milk.
2. La lactancia artificial es más conveniente que la lactancia materna.	2. Formula feeding is more convenient than breast-feeding.
3. El amamantamiento aumenta el vínculo afectivo entre madre e hijo.	3. Breast-feeding increases mother–infant bonding.
4. La leche materna carece de hierro.	4. Breast milk is lacking in iron.
5. Los bebés que se alimentan con leche artificial son más propensos a estar sobrealimentados que los alimentados con leche materna.	5. Formula fed babies are more likely to be overfed than breast-fed babies.
6. La lactancia artificial es la mejor elección si la madre planea trabajar fuera de casa.	6. Formula feeding is the better choice if a mother plans to work outside the home.
7. Las madres que alimentan a sus bebés con leche artificial se pierden uno de los grandes placeres de la maternidad.	7. Mothers who formula feed miss one of the great joys of motherhood.
8. Las madres no deberían amamantar en sitios públicos, como restaurantes.	8. Women should not breast-feed in public places such as restaurants.
9. Los bebés que se alimentan con leche materna son más sanos que los bebés que se alimentan con leche artificial.	9. Babies fed breast milk are healthier than babies who are fed formula.
10. Los bebés alimentados con leche materna son más propensos a estar sobrealimentados que los alimentados con leche artificial.	10. Breast-fed babies are more likely to be overfed than formula fed babies.
11. Los padres se sienten excluidos si la madre amamanta.	11. Fathers feel left out if a mother breast-feeds.
12. La leche materna es la alimentación ideal para los bebés.	12. Breast milk is the ideal food for babies.
13. La leche materna se digiere más fácilmente que la leche artificial.	13. Breast milk is more easily digested than formula.
14. La leche artificial es tan saludable para el niño como la leche materna.	14. Formula is as healthy for an infant as breast milk.
15. La lactancia materna es más conveniente que la lactancia artificial.	15. Breast-feeding is more convenient than formula feeding.
16. La leche materna es más barata que la leche artificial.	16. Breast milk is less expensive than formula.
17. Una madre que bebe alcohol ocasionalmente no debería amamantar a su bebé.	17. A mother who occasionally drinks alcohol should not breast-feed her baby.

© Research Group GRINCAR, MC Suárez-Cotelo: translated with kind permission of John Wiley and Sons.

## Appendix B

**Table A2:** Score distribution of the Spanish (IIFAS-S) version of the Iowa Infant Feeding Attitude Scale (IIFAS).

	IIFAS-S
Mean	69.34
Median	70.00
SE	7.82
Skewness	-0.62
Kurtosis	0.33
Range	39

## Appendix D

**Table A3:** Evaluation of reliability and internal consistency of the total IIFAS-S score (*n* = 118).

Item Variable ^a^	Cronbach’s Alpha If Item Is Deleted	Item-Total Correlations
1 ^b^	0.774	0.403
2 ^b^	0.765	0.597
3	0.772	0.422
4 ^b^	0.787	0.428
5	0.761	0.517
6 ^b^	0.776	0.505
7	0.773	0.540
8 ^b^	0.789	0.477
9	0.756	0.511
10 ^b^	0.784	0.528
11 ^b^	0.787	0.527
12	0.770	0.452
13	0.764	0.507
14 ^b^	0.761	0.543
15	0.762	0.538
16	0.788	0.432
17 ^b^	0.796	0.533

^a^ The name of the items can be found in Table A1; ^b^ It was reversed when calculating the score.

## References

[1] National Institute for Public Health and the Environment Health effects of Breastfeeding: An Update. http://www.rivm.nl/dsresource?objectid=rivmp:276940&type=org&disposition=inline&ns_nc=1.

[2] World Health Organization Global Strategy for Infant and Young Child Feeding. http://whqlibdoc.who.int/publications/2003/9241562218.pdf?ua=1.

[3] Ministerio de Sanidad, Servicios Sociales e Igualdad Informe anual del Sistema Nacional de Salud 2012. http://www.msssi.gob.es/estadEstudios/estadisticas/sisInfSanSNS/tablasEstadisticas/InfSNS2012.htm.

[4] Dungy C.I., Losch M., Russell D. (1994). Maternal attitudes as predictors of infant feeding decisions. J. Assoc. Acad. Minor. Phys..

[5] Chen C.H., Chi C.S. (2003). Maternal intention and actual behavior in infant feeding at one month postpartum. Acta Paediatr. Taiwan.

[6] De la Mora A., Russell D.W. (1999). The Iowa infant feeding attitude scale: Analysis of reliability and validity. J. Appl. Soc. Psychol..

[7] Al-Akour N.A., Khassawneh M.Y., Khader Y.S., Ababneh A.A., Haddad A.M. (2010). Factors affecting intention to breastfeed among Syrian and Jordanian mothers: A comparative cross-sectional study. Int. Breastfeed. J..

[8] Charafeddine L., Tamim H., Soubra M., De la Mora A., Nabulsi M., Research and Advocacy Breastfeeding Team (2016). Validation of the Arabic version of the Iowa Infant Feeding Attitude Scale among Lebanese women. J. Hum. Lact..

[9] Dungy C.I., Losch M.E., Russell D., Romitti P., Dusdieker L.B. (1997). Hospital infant formula discharge packages. Do they affect the duration of breast-feeding?. Arch. Pediatr. Adolesc. Med..

[10] Dungy C.I., McInnes R.J., Tappin D.M., Wallis A.B., Oprescu F. (2008). Infant feeding attitudes and knowledge among socioeconomically disadvantaged women in Glasgow. Matern. Child Health J..

[11] Giglia R.C., Binns C.W., Alfonso H.S., Zhao Y. (2007). Which mothers smoke before, during and after pregnancy?. Public Health.

[12] Ho Y.J., McGrath J.M. (2011). A Chinese version of Iowa Infant Feeding Attitude Scale: Reliability and validity assessment. Int. J. Nurs. Stud..

[13] Kavanagh K.F., Lou Z., Nicklas J.C., Habibi M.F., Murphy L.T. (2012). Breastfeeding knowledge, attitudes, prior exposure, and intent among undergraduate students. J. Hum. Lact..

[14] Lau Y., Htun T.P., Lim P.I., Ho-Lim S.S., Klainin-Yobas P. (2016). Psychometric properties of the Iowa Infant Feeding Attitude Scale among a multiethnic population during pregnancy. J. Hum. Lact..

[15] Mahoney M.C., James D.M. (2000). Predictors of anticipated breastfeeding in an urban, low-income setting. J. Fam. Pract..

[16] Marrone S., Vogeltanz-Holm N., Holm J. (2008). Attitudes, knowledge, and intentions related to breastfeeding among university undergraduate women and men. J. Hum. Lact..

[17] Mulcahy H., Phelan A., Corcoran P., Leahy-Warren P. (2012). Examining the breastfeeding support resources of the public health nursing services in Ireland. J. Clin. Nurs..

[18] Nanishi K., Jimba M. (2014). Reliability and validity of the Japanese version of the Iowa Infant Feeding Attitude Scale: A longitudinal study. J. Hum. Lact..

[19] Scott J.A., Binns C.W., Oddy W.H., Graham K.I. (2006). Predictors of breastfeeding duration: Evidence from a cohort study. Pediatrics.

[20] Scott J.A., Shaker I., Reid M. (2004). Parental attitudes toward breastfeeding: Their association with feeding outcome at hospital discharge. Birth.

[21] Shaker I., Scott J.A., Reid M. (2004). Infant feeding attitudes of expectant parents: Breastfeeding and formula feeding. J. Adv. Nurs..

[22] Sittlington J., Stewart-Knox B., Wright M., Bradbury I., Scott J.A. (2007). Infant-feeding attitudes of expectant mothers in Northern Ireland. Health Educ. Res..

[23] Tappin D., Britten J., Broadfoot M., McInnes R. (2006). The effect of health visitors on breastfeeding in Glasgow. Int. Breastfeed. J..

[24] Tomas-Almarcha R., Oliver-Roig A., Richart-Martinez M. (2016). Reliability and validity of the reduced Spanish version of the Iowa Infant Feeding Attitude Scale. J. Obstet. Gynecol. Neonatal Nurs..

[25] Tuthill E.L., Butler L.M., McGrath J.M., Cusson R.M., Makiwane G.N., Gable R.K., Fisher J.D. (2014). Cross-cultural adaptation of instruments assessing breastfeeding determinants: A multi-step approach. Int. Breastfeed. J..

[26] Twells L.K., Midodzi W.K., Ludlow V., Murphy-Goodridge J., Burrage L., Gill N., Halfyard B., Schiff R., Newhook L.A. (2016). Assessing infant feeding attitudes of expectant women in a provincial population in Canada: Validation of the Iowa Infant Feeding Attitude Scale. J. Hum. Lact..

[27] Wallis A.B., Brînzaniuc A., Cherecheş R., Oprescu F., Sirlincan E., David I., Dîrle I.A., Dungy C.I. (2008). Reliability and validity of the Romanian version of a scale to measure infant feeding attitudes and knowledge. Acta Paediatr..

[28] Wilkins C., Ryan K., Green J., Thomas P. (2012). Infant feeding attitudes of women in the United Kingdom during pregnancy and after birth. J. Hum. Lact..

[29] Chambers J.A., McInnes R.J., Hoddinott P., Alder E.M. (2007). A systematic review of measures assessing mothers’ knowledge, attitudes, confidence and satisfaction towards breastfeeding. Breastfeed. Rev..

[30] Ministry of Health, Social Services and Equality Clinical Practice Guideline for Care in Pregnancy and Puerperium. http://www.guiasalud.es/GPC/GPC_533_Embarazo_AETSA_compl_en.pdf.

[31] Kozhimannil K.B., Jou J., Attanasio L.B., Joarnt L.K., McGovern P. (2014). Medically complex pregnancies and early breastfeeding behaviors: A retrospective analysis. PLoS ONE.

[32] Beaton D.E., Bombardier C., Guillemin F., Ferraz M.B. (2007). Recommendations for the Cross-Cultural Adaptation of the DASH & QuickDASH Outcome Measures.

[33] Sperber A.D., Devellis R.F., Boehlecke B. (1994). Cross-cultural translation: Methodology and validation. J. Cross. Cult. Psychol..

[34] DeVellis R.F. (2011). Scale Development: Theory and Applications.

[35] Cronbach L.J. (2004). My current thoughts on coefficient alpha and successor procedures. Educ. Psychol. Meas..

[36] Steinmetz J.P., Brunner M., Loarer E., Houssemand C. (2010). Incomplete psychometric equivalence of scores obtained on the manual and the computer version of the Wisconsin Card Sorting Test?. Psychol. Assess..

[37] Jaju A., Crask M.R. (1999). The perfect design: Optimization between reliability, validity, redundancy in scale items and response rates. Am. Market. Assoc..

[38] Streiner D.L. (1994). Figuring out factors: The use and misuse of factor analysis. Can. J. Psychiatry.

[39] Scott J.A., Kwok Y.Y., Synnott K., Bogue J., Amarri S., Norin E., Gil A., Edwards C.A., Other Members of the INFABIO Project Team (2015). A comparison of maternal attitudes to breastfeeding in public and the association with breastfeeding duration in four European countries: Results of a cohort study. Birth.

[40] Li R., Hsia J., Fridinger F., Hussain A., Benton-Davis S., Grummer-Strawn L. (2004). Public beliefs about breastfeeding policies in various settings. J. Am. Diet. Assoc..

[41] American Academy of Pediatrics Committee on Drugs (2001). Transfer of drugs and other chemicals into human milk. Pediatrics.

[42] Boletín Oficial del Estado. https://www.boe.es/buscar/act.php?id=BOE-A-2015-11430.

[43] Barona-Vilar C., Escribá-Agüir V., Ferrero-Gandía R. (2009). A qualitative approach to social support and breast-feeding decisions. Midwifery.

